# Memantine increases the dendritic complexity of hippocampal young neurons in the juvenile brain after cranial irradiation

**DOI:** 10.3389/fonc.2023.1202200

**Published:** 2023-10-04

**Authors:** Georgios Alkis Zisiadis, Androniki Alevyzaki, Elene Nicola, Carlos F. D. Rodrigues, Klas Blomgren, Ahmed M. Osman

**Affiliations:** ^1^ Department of Women’s and Children’s Health, Karolinska Institutet, Stockholm, Sweden; ^2^ Pediatric Oncology, Karolinska University Hospital, Stockholm, Sweden

**Keywords:** radiotherapy, neurogenesis, neural stem cells, doublecortin, NMDA antagonist

## Abstract

**Introduction:**

Cranial irradiation (IR) negatively regulates hippocampal neurogenesis and causes cognitive dysfunctions in cancer survivors, especially in pediatric patients. IR decreases proliferation of neural stem/progenitor cells (NSPC) and consequently diminishes production of new hippocampal neurons. Memantine, an NMDA receptor antagonist, used clinically to improve cognition in patients suffering from Alzheimer’s disease and dementia. In animal models, memantine acts as a potent enhancer of hippocampal neurogenesis. Memantine was recently proposed as an intervention to improve cognitive impairments occurring after radiotherapy and is currently under investigation in a number of clinical trials, including pediatric patients. To date, preclinical studies investigating the mechanisms underpinning how memantine improves cognition after IR remain limited, especially in the young, developing brain. Here, we investigated whether memantine could restore proliferation in the subgranular zone (SGZ) or rescue the reduction in the number of hippocampal young neurons after IR in the juvenile mouse brain.

**Methods:**

Mice were whole-brain irradiated with 6 Gy on postnatal day 20 (P20) and subjected to acute or long-term treatment with memantine. Proliferation in the SGZ and the number of young neurons were further evaluated after the treatment. We also measured the levels of neurotrophins associated with memantine improved neural plasticity, brain-derived neurotrophic factor (BDNF) and nerve growth factor (NGF).

**Results:**

We show that acute intraperitoneal treatment with a high, non-clinically used, dose of memantine (50 mg/kg) increased the number of proliferating cells in the intact brain by 72% and prevented 23% of IR-induced decrease in proliferation. Long-term treatment with 10 mg/kg/day of memantine, equivalent to the clinically used dose, did not impact proliferation, neither in the intact brain, nor after IR, but significantly increased the number of young neurons (doublecortin expressing cells) with radial dendrites (29% in sham controls and 156% after IR) and enhanced their dendritic arborization. Finally, we found that long-term treatment with 10 mg/kg/day memantine did not affect the levels of BDNF, but significantly reduced the levels of NGF by 40%.

**Conclusion:**

These data suggest that the enhanced dendritic complexity of the hippocampal young neurons after treatment with memantine may contribute to the observed improved cognition in patients treated with cranial radiotherapy.

## Introduction

Adult hippocampal neurogenesis persists in the rodent brain, from neural stem/progenitor cells (NSPC) residing in the subgranular zone (SGZ). They divide frequently and differentiate into glial or neuronal precursors (1). More than 50% of the newborn cells undergo apoptosis shortly after exiting the cell cycle, and a subset of the neuronal progenitors survives and gives rise to new granule neurons (1–3). Newborn neuronal progenitors acquire distinct developmental morphologies during the process of maturation (4) and transiently express the microtubule-associated protein doublecortin (DCX) along their immature stages, often referred to as young or immature neurons (5, 6). Within the first two weeks after birth, newborn granule neurons acquire a morphology resembling the mature neurons, characterized by a radial projection of the dendritic processes towards the molecular layer and axons extending to the *cornu ammonis 3* region (CA3) (7). They become fully mature within 4-6 weeks after birth (1, 8). In rodents, newborn granule neurons have been shown to contribute to certain cognitive tasks such as pattern separation and maintenance of memory functions (1, 9, 10). In humans, persistence of postnatal hippocampal neurogenesis remains debated, however, there is a consensus that DCX-expressing young neurons are detectable during the first decade of childhood, and whether these cells are newborn neurons or late developing neurons is still an active area of research (11–13).

Several factors regulate NSPC proliferation and neurogenesis. Negative regulators include stress, inflammation, aging and radiation (14–16). Cranial irradiation (IR) is an effective tool to treat brain tumors, but it results in long-term neurocognitive sequelae in cancer survivors, especially pediatric patients (17, 18). Accumulating evidence suggests IR-induced loss of hippocampal neurogenesis is one of the mechanisms behind impaired cognition observed in rodent models and IR-treated patients (19–21). In rodents, several reports have linked NSPC dysfunction after IR and the resultant cognitive impairments to microglial activation and induction of neuroinflammation (16, 22–24). These cognitive deficits are more severe in females, both in animal models and patients (19, 25).

Positive regulators of neurogenesis include physical activity, environmental enrichment, and pharmacological agents such as memantine (14, 26). Memantine is an uncompetitive and low-affinity N-methyl-D-aspartate glutamate (NMDA) receptor antagonist that exerts neuroprotection properties, increases synaptic plasticity (27–29), and is currently approved in clinical practice in the treatment of Alzheimer´s disease patients (30, 31). In animal models, memantine has been shown to substantially increase NSPC proliferation in the intact and diseased brain, that in turn leads to increased neurogenesis and to improved cognition (26, 32–35). Previous studies have linked the memantine-induced enhanced neurogenesis and neural plasticity to increased production of the neurotrophins brain-derived neurotrophic factor (BDNF) and the nerve growth factor (NGF) (32, 36).

Cancer survivors subjected to cranial IR and treated with memantine demonstrated improved cognition (37, 38). For pediatric patients, there are ongoing clinical trials assessing the feasibility of leveraging memantine to reduce cognitive impairment after radiotherapy for central nervous system tumors (ClinicalTrials.gov; identifiers: NCT03194906 and CT04217694). To date, the underlying mechanisms by which memantine improves cognition after IR are yet not fully understood. Recent studies in rodents have shown that pre-treatment with memantine improves synaptic plasticity after IR (39, 40). Whether memantine influences hippocampal neurogenesis after IR is yet limited to one study performed in an adult mouse IR model (41). Given that the rate of hippocampal neurogenesis is considerably higher in young animals (42, 43); leading to more severe injuries in the young, still developing brain (44), we set out to investigate whether treatment with memantine improves proliferation in the SGZ and restores the IR-induced reduction in the number of hippocampal young neurons after IR in the juvenile brain. We tested different treatment paradigms administered after subjecting the mice to IR. We show that treatment with a high, not clinically used, dose of memantine significantly increased proliferation in the SGZ in the intact brain and reduced the IR-induced loss of proliferation, but was not sufficient to restore the IR-induced reduction in the number of the young neurons in the hippocampus. Continuous supplementation with a lower, equivalent to clinically used, dose did not promote proliferation in the SGZ neither in the intact, nor the irradiated brain, but rather enhanced the dendric complexity of the young neurons in the GCL in the dentate gyrus of the hippocampus. Finally, we found that continuous supplementation with a low, clinically relevant, dose of memantine did not affect the levels of BDNF, but significantly reduced the levels of NGF.

## Materials and methods

### Animals

Female C57Bl6/J mice (Charles River, Sulzfeld, Germany) were used in all studies, as IR-induced cognitive deficits are more severe in females, both in animal models and patients (19, 25). Mice were housed and maintained in a controlled 12/12 h light/dark cycle, 20°C ambient temperature and 80% relative humidity, with access to food and water *ad libitum*. All the experimental procedures were carried out according to the European and Swedish animal welfare regulations approved by the northern Stockholm ethical committee (application nr. N248/13). Litter-mate mice were randomly divided into 4 treatment groups: sham controls (SH) receiving vehicle; SH treated with memantine; IR receiving vehicle; IR treated with memantine.

### Irradiation procedure

Twenty-day-old (P20) mice were anesthetized with isoflurane (5% and 1.5% for induction and maintenance, respectively) in a mixture of oxygen/air (1:1) at a flow rate of 0.3 L/min. A single dose of 6 Gy was administered, at a dose rate of 0.73 Gy/min using X-RAD 320 X-ray machine (PXi Precision X-ray, North Branford, CT, USA). SH animals were only anesthetized with isoflurane for a similar time as the IR mice. Using the linear quadratic model and an α/β ratio of 3 for late effects in normal brain tissue, the acute exposure of 6 Gy used in this study is equivalent to 11 Gy when delivered in 2 Gy fractions (45), that is sufficient to impair neurogenesis and worsen neurocognitive performance in mice (46, 47).

### Memantine preparation and administration

**Figure 1:**
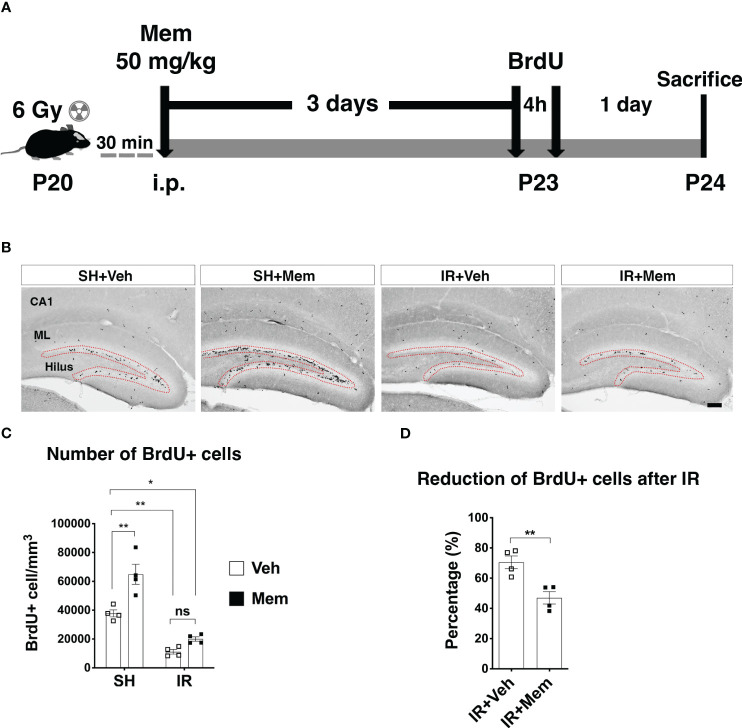
Treatment with a single i.p. injection of a high dose of memantine (Mem) rescues proliferation in the SGZ after IR. **(A)** Scheme for the experimental design. **(B)** Representative images for BrdU+ cells in the hippocampus of the treatment groups. BrdU+ cells were only quantified in the subgranular zone (SGZ; red dotted area) of the dentate gyrus where neural stem/progenitor cells (NSPCs) and their progenies reside. Scalebar = 100 μm. SH, sham; Veh, Vehicle; Mem, Memantine; IR, irradiated; CA1, *Cornu ammonis* 1; ML, Molecular layer. **(C)** Quantification of BrdU+ cells in the SGZ. n = 4 per group. Data represent mean ± SEM. Two-way ANOVA with Tukey’s *post hoc* test for multiple comparisons. *P < 0.03, **P < 0.003. ns, not significant. **(D)** Percentage of reduction of BrdU+ cells in the SGZ after IR compared to sham control treated with Veh. n = 4. Data represent mean ± SEM. Unpaired *t*-test **P = 0.007.

**Figure 2:**
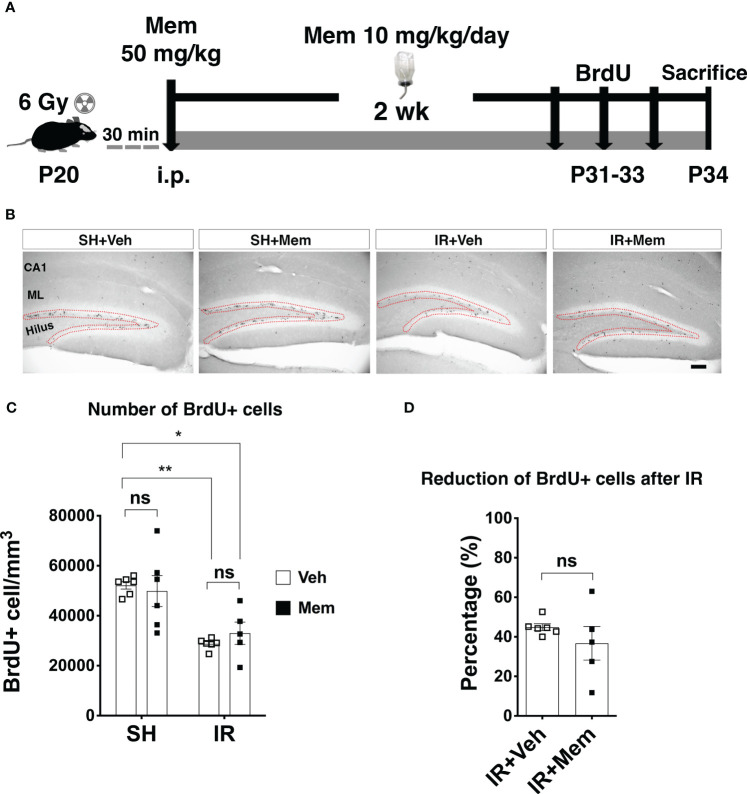
Long-term oral treatment with a low dose of Mem does not impact proliferation in the SGZ. **(A)** Scheme for the experimental design. **(B)** Representative images for BrdU+ cells in the hippocampus of the treatment groups. BrdU+ cells were only quantified in SGZ (red dotted area). Scalebar = 100 μm. **(C)** Quantification of BrdU+ cells in the SGZ. SH + Veh n = 6; SH + Mem n = 6; IR + Veh n = 6; IR + Mem n = 5. Data represent mean ± SEM. Two-way ANOVA with Tukey’s *post hoc* test for multiple comparisons. *P < 0.05, **P < 0.005. ns, not significant. **(D)** Percentage of reduction of BrdU+ cells in the SGZ after IR compared to sham control treated with Veh. IR + Veh n = 6; IR + Mem n = 5. Data represent mean ± SEM. Unpaired *t*-test, ns, not significant.

Memantine hydrochloride (Sigma-Aldrich #M9292) was dissolved in saline solution (0.9% sodium chloride) to achieve a concentration of 10 mg/ml and stored at +4°C. For all treatment paradigms, mice received a single intraperitoneal (i.p.) injection of a loading dose of 50 mg/kg (high dose) memantine or an equal volume of vehicle (saline) administered within 30 min after IR, followed by continuous treatment with 10 mg/kg/day (low dose) of memantine for 2 weeks supplied in the drinking water (referred to as long-term treatment). This dose achieves a steady-state plasma concentration of ~0.5 μM equivalent to the therapeutic levels achieved when patients are treated with 20 mg/day of memantine (35, 48), referred to as a clinically relevant dose henceforth. Vehicle-treated animals were supplied with regular water. For the intermittent treatment, mice received the loading dose of memantine, followed by a daily single i.p. injections of 10 mg/kg memantine or an equal volume of saline (**Figures 1A**, **2A**, **Supplementary Figures 1A**, **2A**).

### 5-bromo-2’-deoxyuridine administration

For all experiments, BrdU (SigmaAldrich #B5002) was injected i.p. at a dose of 50 mg/kg to label proliferating cells, including NSPCs and their prolific progenies residing in the SGZ (49, 50). To assess proliferation in the SGZ after treatment with a single high dose of memantine, animals received 2 injections of BrdU (4 h apart) three days after treatment with memantine (**Figure 1A**). To assess proliferation in the SGZ after long-term oral treatment with a low dose of memantine, animals received BrdU during the last three days of treatment with memantine, single injection a day (**Figure 2A**).

### Tissue preparation

Mice were transcardially perfused with saline and the brains were collected. Brain hemispheres were separated, and the right hemisphere (without cerebellum or the olfactory bulb) was placed into a 2 ml microcentrifuge tube, snap-frozen on dry ice and stored at -80°C and further processed for protein extraction and ELISA (see below). The left hemisphere was stored in 4% paraformaldehyde (PFA, Histolab Products AB, Sweden), and fixed for 48 h at +4°C. Samples were then dehydrated in 30% sucrose (Sigma #S7903; made in 0.1 M phosphate buffer) for at least 2 days and processed for immunohistochemistry (see below).

### Immunohistochemistry and immunofluorescence

Twenty-five μm thick sagittal free-floating sections were cut in a 1:12 series interval using a sliding microtome (Leica SM2010R) and stored in 2 ml Eppendorf tubes containing a cryoprotectant solution (25% glycerol, 25% ethylene glycol in 0.1M phosphate buffer) and kept at +4°C.

After several washes with 1× Tris-buffered saline (TBS), samples were incubated in sodium citrate solution (NaCi, 10 mM, pH 6.0) for 30 min at 80°C for antigen retrieval. When immunoperoxidase staining used, sections were incubated in a 0.6% hydrogen peroxide (H_2_O_2_) solution for 30 min to quench the endogenous peroxidase. Sections were incubated in a blocking solution containing 5% donkey serum (Jackson ImmunoResearch Laboratories, West Grove, PA) and 0.1% Triton X-100 for 60 min at room temperature to prevent the non-specific-binding of the antibodies. Sections were then incubated with the following primary antibodies at 4°C for 48 - 72 h: rat anti-BrdU (1:500, Serotec #OBT0030G), goat or mouse anti-DCX (1:500; Santa Cruz Biotechnology #sc-8066 or #sc-271390, respectively), rabbit anti-DCX (1:500; Abcam #Ab207175). Sections were rinsed with TBS and incubated with the appropriate secondary antibodies for 1 h (for immunoperoxidase staining) or 2 h (for immunofluorescence) at room temperature. The following secondary antibodies were used: Biotinylated donkey anti-rat IgG (#712-066-150), biotinylated donkey anti-goat IgG (#705-065-147) and biotinylated donkey anti-mouse IgG (#715-065-151), (all from ImmunoResearch Laboratories; 1:1000), Alexa-555 donkey anti-rabbit IgG (1:1,000; Molecular probes/Life Technologies #A31572). Hoechst 33342 (Molecular Probes/Life Technologies #H3570) was used as a nuclear counterstain when fluorescence staining was used. To visualize the immunoperoxidase staining, sections were incubated for 1 h at room temperature in avidin-biotin solution (1:100; Vectastain ABC Elite kit, Vector Laboratories, Burlingame, CA). The color precipitate was developed with a solution containing H_2_O_2_, nickel chloride, and 3-3´diaminobenzidine tetrahydrochloride (DAB; 1:100; Saveen Werner AB, Malmö, Sweden). Sections were mounted into slides, dehydrated with NeoClear (Merck #1.09843.5000, Germany) and coverslipped using NeoMount mounting medium (Merck #1.09016.0500, Germany). ProLong Gold anti-fade reagent (Molecular probes/Life Technologies #P36930) was used as mounting medium for the fluorescent staining.

### Microscopy and cell quantification

The numbers of BrdU and DCX positive cells (BrdU+ and DCX+, respectively) were quantified in two independent sets of sections where the immunoreactivity of each marker was visualized using the immunoperoxidase and DAB staining method (described above). Quantification was performed using a bright field microscope (AxioImager M2; Carl Zeiss microscopy, Germany) equipped with the StereoInvestigator software (MicroBrightField Inc.). The analyses were performed in sections containing the dorsal hippocampus spaced 150 μm apart (*i.e.* every sixth series). The area of interest was traced using the 10× objective lens and quantification was performed using the 20× objective lens. The BrdU+ cells were only quantified in the subgranular zone (SGZ) where NSPCs and their progenies reside. The DCX+ cells were quantified in both the SGZ and the granule cell layer (GCL). For quantification of DCX+ cells, the light condenser below the slide holder was raised to its maximum level, the light illumination was turned on to its maximum, and the exposure time was adjusted in the StereoInvestigator software to allow visualization of the cell bodies of DCX+ cells. Cells were considered for quantification only when the cell body was clearly defined. For both analyses, the cell density was determined by dividing the total number of quantified cells by the volume of the tissue where the cells were quantified. The volume of the tissue was determined by multiplying the traced area by the tissue thickness and the series interval (*i.e.* the area × 25 × 6).

### Analysis of dendritic complexity of young neurons

**Figure 3:**
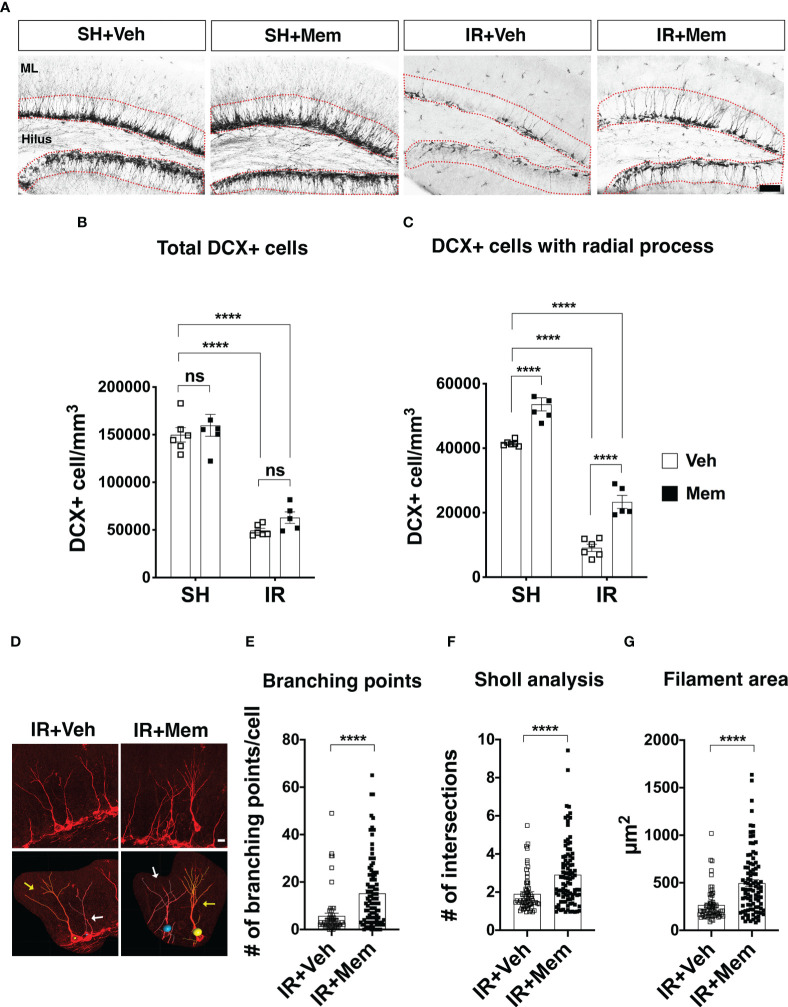
Long-term oral treatment with a low dose of Mem increases the number of DCX+ cells with radial processes and their dendritic complexity. **(A)** Representative images for DCX+ cells in the dentate gyrus of the treatment groups. DCX+ cells were quantified in SGZ and the granule cell layer (GCL) (red dotted area). Scalebar = 50 μm. **(B)** Quantification of total DCX+ cells. SH + Veh n = 6; SH + Mem n = 6; IR + Veh n = 6; IR + Mem n = 5. Data represent mean ± SEM. Two-way ANOVA with Tukey’s *post hoc* test for multiple comparisons. ****P < 0.0001. ns = not significant. **(C)** Quantification of DCX+ cells with radial processes projecting towards the ML. SH + Veh n = 6; SH + Mem n = 6; IR + Veh n = 6; IR + Mem n = 5. Data represent mean ± SEM. Two-way ANOVA with Tukey’s *post hoc* test for multiple comparisons. ****P < 0.0001. **(D)** Representative images of filament tracing performed on DCX+ cells with radial processes from IR animals treated with either Veh or Mem. In the lower panel, cells with yellow tracing (yellow arrow) display no overlap with a nonboring cell that considered for analysis. By contrast, cells with the white tracing (white arrows) display an overlap with a neighboring cell, which excluded from the analysis. Scalebar = 10 μm. **(E-G)** Analysis of dendritic complexity of the DCX+ cells in the IR animals at 2 wks after treatment with Veh (n = 64 cells) or Mem (n = 95 cells). **(E)** Quantification of the branching points. **(F)** Scholl analysis for the number of intersections in the dendritic tree. **(G)** Measurement of the area covered by the dendritic tree. Data represent mean ± SEM. Unpaired *t*-test. ****P < 0.0001.

To analyze the arborization and dendritic complexity of the young neurons after treatment with memantine, immunofluorescence staining for DCX was performed (described above) and z-stack images were acquired from two sections per animal containing the dorsal hippocampus using a laser scanning confocal microscopy (LSM700, Carl Zeiss, Germany). Images were processed for reconstruction and filament tracing on Imaris software (Imaris V 9.6). Only individual cells that could be traced with sufficient certainty there was no overlap with processes from neighboring cells were consider for analysis. Thus, this analysis was only performed in the irradiated animals, as it was challenging to trace individual cells in the sham control animals without overlap from neighboring cells (**Figures 3D**; **Supplementary Figure 3**). A total of 64 and 95 DCX+ cells with radial processes were reconstructed from IR + vehicle or IR + memantine animals, respectively, and analyzed for the number of branching points, Sholl intersections and the area covered by the dendric processes.

### Protein extraction and ELISA

Ice-cold protein extraction buffer (50 mM Tris-HCl; Sigma #T1503, 100 mM NaCl; Sigma, #S7653; 5 mM EDTA, Sigma # E5134 and 1 mM EGTA, Sigma #E3889) supplemented with protease inhibitor cocktail (Roche #11836170001) and phosphatase inhibitors (Roche #04906837001) were added to the frozen tissue and homogenized with a sonicator. Samples were then centrifuged for 10 min in 4°C at 10,000×g and the supernatant was transferred into 0.5 ml tubes and stored at -80°C. The total protein concentration was determined using the Pierce BCA protein assay kit (Thermo Fischer Scientific #23225).

The levels of BDNF were measured using a BDNF ELISA kit (Abcam #ab212166), and NGF was measured using mouse a NGF ELISA kit (LifeSpan Biosciences #LS-F2522). The assays were performed according to the manufacturer’s instructions.

### Statistical analysis

The statistical analysis was performed using GraphPad Prism software (GraphPad Software, Inc., San Diego, CA, USA). Data are presented as mean ± SEM. Two-way ANOVA with Tukey´s *post hoc* test for multiple comparisons was used when comparing multiple treatment groups. Unpaired *t*-test was applied when comparing two treatment groups. Statistical significance was considered when *P* < 0.05.

## Results

### A single, high dose of memantine increases proliferation in the SGZ in the intact brain and partially ameliorates the IR-induced reduction of proliferation

To investigate whether treatment with memantine restores proliferation in the SGZ after IR, we first treated the mice with a single i.p. injection of 50 mg/kg, a dose approximately 10-fold higher than clinical therapeutic doses in humans (referred to as non-clinically relevant henceforth), but previously reported to increase NSPC proliferation in the intact adult brain (26, 33). Sham controls (SH) and IR animals were treated with either memantine or vehicle (0.9% sodium chloride) immediately after whole-brain IR. Three days later, animals received two injections of the thymidine analog BrdU to label dividing cells, including NSPCs and their proliferating progenies in the SGZ (49, 50), given 4 h apart, and the animals were sacrificed 1 day later (**Figure 1A**). In the hippocampus, BrdU positive (BrdU+) cells were observed in the SGZ, where NSPCs and their progenies reside, as well as in areas outside the neurogenic zone, both in SH and IR animals, such as the hilus, the molecular layer and the of the *cornu ammonis* 1 region (CA1) (**Figure 1B**), where oligodendrocyte precursors or microglia proliferate in the postnatal hippocampus (51, 52). As the purpose of this study was to evaluate the effect of memantine on proliferation of potential NSPCs and their progenies, we analyzed the number of BrdU+ cells in the SGZ. In SH animals, memantine treatment resulted in a 72% significant increase in the number of BrdU+ cells in the SGZ compared to those receiving vehicle (**Figures 1B, C**). As expected, IR significantly reduced the number of BrdU+ cells compared to SH controls (**Figures 1B, C**), but when we compared the percentage of the decreased proliferation in the SGZ between IR animals receiving vehicle with those treated with memantine (both compared with SH control receiving only vehicle as baseline control), we found that treatment with memantine significantly preserved 23% of proliferation in the SGZ (**Figure 1D**). This indicates that memantine at a higher dose promotes proliferation in the SGZ in the intact brain, and partially preserves their proliferation after IR.

### Long-term treatment with a clinically relevant dose of memantine does not alter proliferation, neither in the intact brain, nor after IR

Next, we wanted to investigate whether maintaining the treatment with a clinically used dose of memantine would rescue proliferation in the SGZ after IR. SH and IR animals first received a loading dose of 50 mg/kg of memantine or vehicle immediately after whole-brain IR, followed by continuous treatment with 10 mg/kg/day of memantine for 2 weeks supplied in the drinking water. This dose achieves a steady-state plasma concentration of ~0.5 μM equivalent to clinical therapeutic levels achieved when patients are treated with 20 mg/day of memantine (35, 48), referred to as a clinically relevant dose henceforth. Vehicle-treated animals were provided regular drinking water (**Figure 2A**). BrdU labeling of proliferating cells was performed during the last 3 days of the treatment period (**Figure 2A**). We found that continuous treatment with a low dose of memantine did not increase the number of BrdU+ cells in the SGZ, neither in SH, nor after IR (**Figures 2B, C**), and did not rescue the decreased proliferation in the SGZ after IR (**Figure 2D**). These results indicate that long-term oral treatment with a clinically relevant dose of memantine did neither impact proliferation in the SGZ in the intact brain, nor improved IR-induced reduction of proliferation in the SGZ.

### Long-term oral treatment with a clinically relevant dose of memantine does not increase the number of young neurons, but enhances their arborization

We wanted to investigate whether maintaining the treatment with a clinically relevant dose of memantine impacts the number of the young neurons in the GCL. Animals were treated with memantine for 2 weeks (**Figure 2A**), and the young neurons were identified by expression of DCX (DCX+) cells (5). As expected, the number of DCX+ cells significantly dropped after IR (67% in IR + vehicle and 58% in IR + memantine, compared to SH + vehicle as baseline control) (**Figures 3A, B**). Long-term oral treatment with a low dose of memantine did not increase the number of DCX+ cells, neither in SH, nor IR animals (**Figures 3A, B**; however, it significantly increased the number of DCX+ cells with radial processes projecting towards the molecular layer, both in SH (29%) and IR (156%) animals (**Figures 3A, C**). To exclude that this effect was because the loading dose (50 mg/kg) administered initially after IR, we analyzed the total number of DCX+ cells and DCX+ cells with radial processes 2 weeks after receiving only a single i.p. injection of 50 mg/kg or vehicle, and we found comparable numbers between the treatment groups (**Supplementary Figures 1A-C**). Also, we did not observe an increase in the total number of DCX+ cells or those with radial processes when the animals received an intermittent treatment with daily i.p. injections of 10 mg/kg of memantine for 2 weeks (**Supplementary Figures 2A-C**). We next analyzed whether long-term oral treatment with memantine impacts young neuron plasticity by performing filament tracing of the dendritic processes of DCX+ cells in the IR animals, as it is feasible to trace individual cells due to the low number of cells remaining after IR, thus avoiding the overlap with the neighboring cells that often occurs in the SH animals (**Figure 3D**; **Supplementary Figure 3A**). We found that treatment with memantine significantly increased the number of branching points (3-fold), the Sholl intersections (2-fold) and the filament area coverage (2-fold) (**Figures 3D-G**). Collectively, these data show that long-term oral administration of a clinically relevant dose of memantine does not resuce the IR-induced loss of hippocampal young neurons, but rather promotes their radial projection towards the molecular layer and enhances the arborization and the complexity of their dendric processes after IR.

### Long-term oral treatment with memantine reduces the levels of the NGF

**Figure 4:**
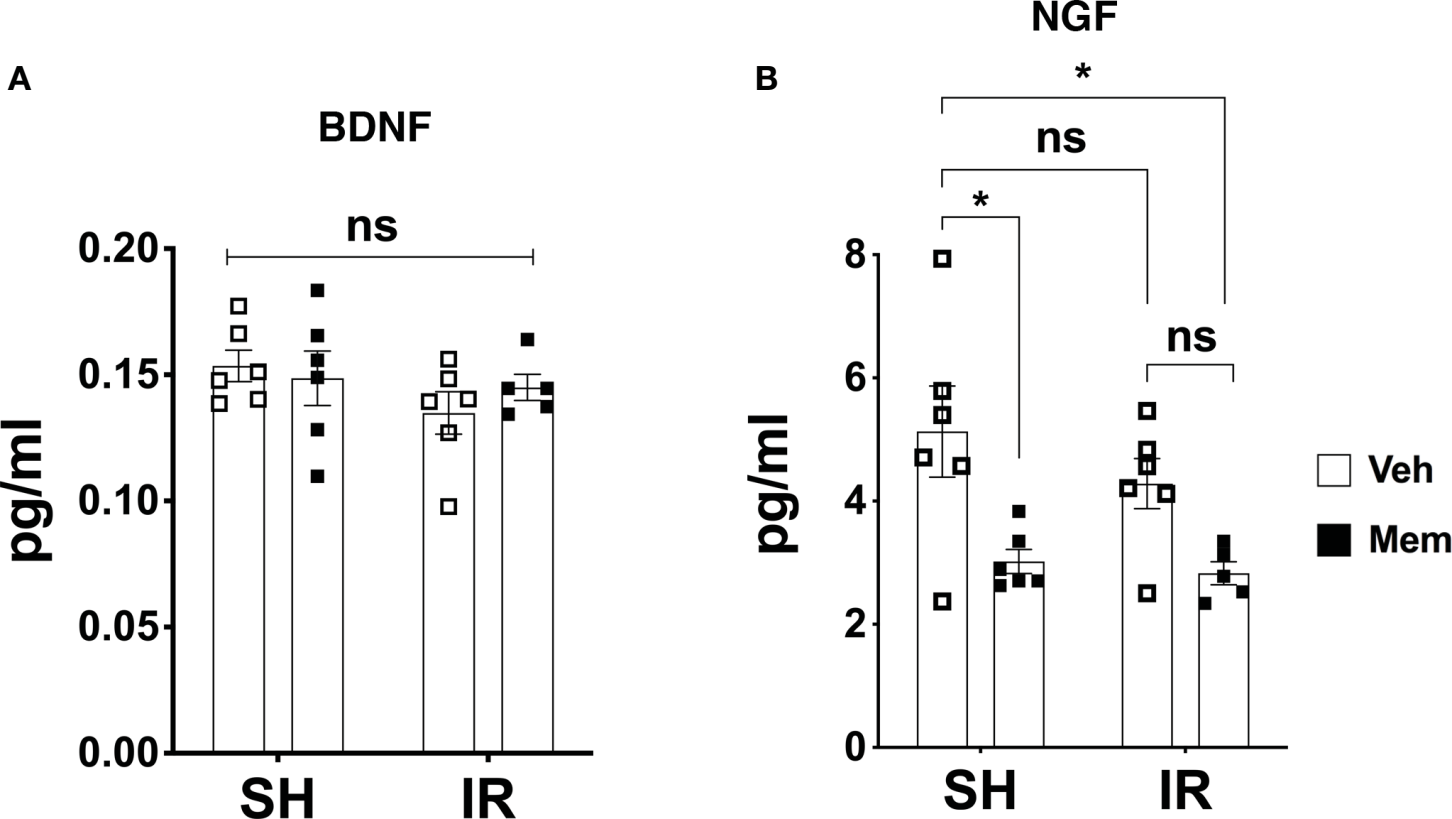
Long-term oral treatment with a low dose of Mem decreases the levels of NGF and does not affect BDNF. **(A)** Protein levels of BDNF in the different treatment groups measured by ELISA. SH + Veh n = 6; SH + Mem n = 6; IR + Veh n = 6; IR + Mem n = 5. Data represent mean ± SEM. Two-way ANOVA with Tukey’s *post hoc* test for multiple comparisons. ns = not significant. **(B)** Protein levels of NGF in the different treatment groups. SH + Veh n = 6; SH + Mem n = 6; IR + Veh n = 6; IR + Mem n = 5. Data represent mean ± SEM. Two-way ANOVA with Tukey’s *post hoc* test for multiple comparisons. *P < 0.03. ns, not significant.

Previous reports have shown that treatment with memantine increases neurogenesis and enhances neural sprouting, and these effects are in part mediated by increased production of the neurotrophins BDNF and NGF (32, 36). Therefore, we wanted to address if the increased dendritic complexity of the young neurons observed after long-term oral treatment with memantine involves these neurotrophins. Therefore, we measured the protein levels of BDNF and NGF in brain homogenates using ELISA. We did not detect any changes in the levels of BDNF, neither after IR, nor after treatment with memantine (**Figure 4A**). Moreover, we found that IR did not affect the levels of NGF, but unexpectedly treatment with memantine resulted in a 40% reduction in its levels, both in SH and IR animals (**Figure 4B**). These data show that the increased dendritic complexity of the young neurons after treatment with memantine is not associated with increased levels of BDNF or NGF, at least at this timepoint of our treatment scheme.

## Discussion

We addressed for the first time the effects of treatment with memantine on cell proliferation in the SGZ and the number of young neurons after IR in the juvenile brain. We show that a high dose of memantine increases proliferation in the SGZ in the intact brain and this partially prevents the loss of proliferation after IR. Long-term oral treatment with a clinically relevant dose of memantine yielding steady-state plasma levels of ~0.5μM (35), equivalent to what is reached with daily treatment with 20 mg/day in patients (48),, does not enhance proliferation in the SGZ, but increases the arborization and the dendritic complexity of the young neurons in the dentate gyrus.

Memantine is used clinically to treat patients with dementia and Alzheimer´s disease (30, 31). Animal models revealed that memantine increases neural plasticity and hippocampal neurogenesis in various pathological conditions known to negatively impact neurogenesis, such as and Alzheimer’s disease and depression, and these effects correlate with improved cognition performance (32–34). In the context of the irradiated brain, as in the treatment of brain tumors, memantine has been tested clinically, and moderately ameliorated the cognitive deficits in cancer survivors (37). In animal models of IR, memantine increased neural plasticity, as evidenced by increased neurite spine densities, and prevented the deterioration of long-term potentiation deterioration in the hippocampus, given that treatment was started before IR (39, 40). Mounting evidence from animal models suggests that cognitive decline resulting from IR is, at least in part, due to loss of hippocampal neurogenesis (15, 16, 19, 20). Previous interventions aimed to increase proliferation and hippocampal neurogenesis, such as treatment with lithium or physical exercise, succeeded to improve cognition in IR animals (46, 53–56). Here, when we tested the effects of memantine, as a potent enhancer of the proliferation in the SGZ, we observed that only a relatively high dose of 50 mg/kg, but not a clinically relevant dose of 10 mg/kg, enhanced proliferation in the intact juvenile brain and ameliorated the degree of proliferation loss in the SGZ caused by IR. However, high and low doses of memantine have been shown to correlate with negative and positive neurocognitive outcomes, respectively (57) suggesting that the enhanced cognitive performance attained after treatment with a low dose of memantine is achieved independently of the effect on proliferation in the SGZ.

IR not only reduces the generation of granule neurons (15, 19, 58, 59), but also worsens the integration of the new neurons, which could partially be rescued by physical exercise, leading to an improved behavioral pattern in rodents after IR (46). Here, we show that long-term treatment with a clinically used dose of memantine drastically increases the number of the young neurons with radial process and boosts the complexity of their dendrites. Yet, whether the observed enhanced dendritic complexity of the young neurons after treatment with memantine would promote their integration into the hippocampal circuitry remains to be functionally tested, but it is tempting to speculate this may contribute to improve cognition, as previously demonstrated in animal studies upon treatment with lower doses of memantine (29, 57).

Contrary to our findings in the juvenile brain, a recent study performed in adult mice (41), treatment with memantine was shown to restore the production of new hippocampal neurons after IR. Animal age is known to regulate the levels of hippocampal neurogenesis and influence the severity of IR-induced brain injury (42–44, 60). In addition to age explaining the different outcomes obtained after IR and memantine treatment, another important factor to consider is the brain irradiation paradigm applied. In the study by Hokama and colleagues (41), mice received a total dose of 10 Gy given in five fractions, 2 Gy per fraction. In our study, mice received a single fraction of 6 Gy. Using the linear quadratic model and an α/β ratio of 3 for late effects in normal brain tissue, the overall biological effective dose and sensitivity of NSPCs in the hippocampus depend on the number of fractions and the dose given per fraction (45, 61).

Several mechanisms are involved in the integration of newborn neurons in the hippocampal circuitry and the promotion of neural plasticity and cognition. Among these are signaling pathways coupled to neurotrophic factors, such as BDNF and NGF (62, 63). Here we did not detect differences in BDNF levels, neither after treatment with memantine, as previously described (28, 32), nor after IR. However, to our surprise, we observed that the levels of NGF were significantly reduced. Despite numerous reports associating increased levels of NGF with greater neuronal sprouting (64–66), modulation of NGF by memantine is as of yet controversial (36, 67), and generation of solid data in this regard remain necessary, particularly to elucidate the surprising correlation between decreased levels of NGF and increased dendritic complexity of young neurons. Although there is a possibility that our treatment and analysis scheme in this study might have not captured the optimal time course at which these trophic factors exert their effects on the young neurons, the molecular mechanisms underlying memantine-induced enhanced neural plasticity may not be limited to BDNF and NGF. Hence generation of transcriptomic or proteomic data over a different time courses after treatment may robustly aid in discovering novel targeted pathways (68).

In summary, we show that long-term oral treatment with a clinically used dose of memantine administered after subjecting the mice to cranial irradiation did not promote proliferation in the SGZ, but rather enhanced the arborization of the dendritic processes of the young neurons. Increased dendritic complexity of young neurons may be one of the mechanisms behind the moderate functional improvement observed in adult cancer survivors treated with memantine (37). A follow up study considering pretreating the mice with memantine before ensuing cranial irradiation is warranted, as it would better represent what is applied in clinical studies (ClinicalTrials.gov; identifiers: NCT03194906 and CT04217694), to evaluate whether this approach would confirm the findings of this study or yield different outcomes. Moreover, treatment paradigms aiming for restoration of hippocampal neurogenesis after IR by long-term treatment with a low dosage of memantine could be combined with other factors known to rescue proliferation of NSPCs and their progenies in the SGZ after IR, such as physical activity (53), lithium (56, 69) or metformin (47). Notably, memantine and lithium are currently approved drugs for treating CNS disorders (30, 31, 70).

## Data availability statement

The raw data supporting the conclusions of this article will be made available by the authors, without undue reservation.

## Ethics statement

The animal study was reviewed and approved by The northern Stockholm ethical committee (application nr. N248/13).

## Author contributions

GZ, AA and EN performed the histological analyses. GZ and CR performed the Imaris analysis. GZ and AO performed the animal experiments. AA and AO performed the ELISA. AO and KB designed the study, interpreted the results and wrote the manuscript. All authors discussed the results and commented on or edited the manuscript.

## Supplementary material

Supplementary Figure 1 Single i.p. injection of a high dose of Mem does not impact on the number of DCX+ cells with radial processes at 2 weeks post-treatment. (**A**) Scheme for the experimental design. wk = week. (**B**) Quantification of total DCX+ cells in the subgranular zone (SGZ) and the granule cell layer in the dentate gyrus of the treatment groups. n = 3 per group. Data represent mean ± SEM. Two-way ANOVA with Tukey’s *post hoc* test for multiple comparisons. *****P* < 0.0001. ns = not significant. (**C**) Quantification of DCX+ cells with radial processes. n = 3 per group. Data represent mean ± SEM. Two-way ANOVA with Tukey’s *post hoc* test for multiple comparisons. *****P* < 0.0001. ns = not significant.

Supplementary Figure 2 Intermittent administration of a low does not no impact on the number of DCX+ cells with radial processes at 2 weeks post-treatment. (**A**) Scheme for the experimental design. wk = week. (**B**) Quantification of total DCX+ cells in the SGZ and the GCL in the dentate gyrus of the treatment groups. SH + Veh n = 4; SH + Mem n = 6; IR + Veh n = 3; IR + Mem n = 4. Data represent mean ± SEM. Two-way ANOVA with Tukey’s *post hoc* test for multiple comparisons. *****P <*0.0001. ns = not significant. (**C**) Quantification of DCX+ cells with radial processes. n = 3-6 per treatment. Two-way ANOVA with Tukey’s *post hoc* test for multiple comparisons. **P* < 0.03*, **P* < 0.005. ns = not significant.

Supplementary Figure 3 Filament tracing of DCX+ cells in sham controls animals. **(A)** Representative immunofluorescent images show filament tracing of reconstructed DCX+ cells in the GCL of SH animals treated with either Veh or Mem. Accurate tracing of individual cells without an overlap with neighboring cells was challenging as demonstrated by the yellow and white tracing in each treatment group. Scalebar = 30 μm.

## References

[1] Aimone JB Li Y Lee SW Clemenson GD Deng W Gage FH. Regulation and function of adult neurogenesis: from genes to cognition. Physiol Rev (2014) 94(4):991–1026. doi: 10.1152/physrev.00004.2014 25287858 PMC4280160

[2] Sierra A Encinas JM Deudero JJ Chancey JH Enikolopov G Overstreet-Wadiche LS. Microglia shape adult hippocampal neurogenesis through apoptosis-coupled phagocytosis. Cell Stem Cell (2010) 7(4):483–95. doi: 10.1016/j.stem.2010.08.014 PMC4008496 20887954

[3] Kuhn HG. Control of cell survival in adult mammalian neurogenesis. Cold Spring Harb Perspect Biol (2015) 7(12). doi: 10.1101/cshperspect.a018895 PMC4665071 26511628

[4] Zhao C Teng EM Summers RG Jr. Ming GL Gage FH. Distinct morphological stages of dentate granule neuron maturation in the adult mouse hippocampus. J Neurosci (2006) 26(1):3–11. doi: 10.1523/JNEUROSCI.3648-05.2006 16399667 PMC6674324

[5] Brown JP Couillard-Despres S Cooper-Kuhn CM Winkler J Aigner L Kuhn HG. Transient expression of doublecortin during adult neurogenesis. J Comp Neurol (2003) 467(1):1–10. doi: 10.1002/cne.10874 14574675

[6] Couillard-Despres S Winner B Schaubeck S Aigner R Vroemen M Weidner N. Doublecortin expression levels in adult brain reflect neurogenesis. Eur J Neurosci (2005) 21(1):1–14. doi: 10.1111/j.1460-9568.2004.03813.x 15654838

[7] Sun GJ Sailor KA Mahmood QA Chavali N Christian KM Song H. Seamless reconstruction of intact adult-born neurons by serial end-block imaging reveals complex axonal guidance and development in the adult hippocampus. J Neurosci (2013) 33(28):11400–11. doi: 10.1523/JNEUROSCI.1374-13.2013 PMC3724551 23843512

[8] Toni N Schinder AF. Maturation and functional integration of new granule cells into the adult hippocampus. Cold Spring Harb Perspect Biol (2015) 8(1):a018903. doi: 10.1101/cshperspect.a018903 26637288 PMC4691791

[9] Folsz O Trouche S Croset V. Adult-born neurons add flexibility to hippocampal memories. Front Neurosci (2023) 17:1128623. doi: 10.3389/fnins.2023.1128623 36875670 PMC9975346

[10] Sahay A Scobie KN Hill AS O'Carroll CM Kheirbek MA Burghardt NS. Increasing adult hippocampal neurogenesis is sufficient to improve pattern separation. Nature (2011) 472(7344):466–70. doi: 10.1038/nature09817 PMC3084370 21460835

[11] Moreno-Jimenez EP Terreros-Roncal J Flor-Garcia M Rabano A Llorens-Martin M. Evidences for adult hippocampal neurogenesis in humans. J Neurosci (2021) 41(12):2541–53. doi: 10.1523/JNEUROSCI.0675-20.2020 PMC8018741 33762406

[12] Sorrells SF Paredes MF Cebrian-Silla A Sandoval K Qi D Kelley KW. Human hippocampal neurogenesis drops sharply in children to undetectable levels in adults. Nature (2018) 555(7696):377–81. doi: 10.1038/nature25975 PMC6179355 29513649

[13] Sorrells SF Paredes MF Zhang Z Kang G Pastor-Alonso O Biagiotti S. Positive controls in adults and children support that very few, if any, new neurons are born in the adult human hippocampus. J Neurosci (2021) 41(12):2554–65. doi: 10.1523/JNEUROSCI.0676-20.2020 PMC8018729 33762407

[14] Braun SM Jessberger S. Adult neurogenesis: mechanisms and functional significance. Development (2014) 141(10):1983–6. doi: 10.1242/dev.104596 24803647

[15] Bostrom M Kalm M Karlsson N Hellstrom Erkenstam N Blomgren K. Irradiation to the young mouse brain caused long-term, progressive depletion of neurogenesis but did not disrupt the neurovascular niche. J Cereb Blood Flow Metab (2013) 33(6):935–43. doi: 10.1038/jcbfm.2013.34 PMC3677115 23486289

[16] Monje ML Mizumatsu S Fike JR Palmer TD. Irradiation induces neural precursor-cell dysfunction. Nat Med (2002) 8(9):955–62. doi: 10.1038/nm749 12161748

[17] Makale MT McDonald CR Hattangadi-Gluth JA Kesari S. Mechanisms of radiotherapy-associated cognitive disability in patients with brain tumours. Nat Rev Neurol (2017) 13(1):52–64. doi: 10.1038/nrneurol.2016.185 27982041 PMC5805381

[18] Han JW Kwon SY Won SC Shin YJ Ko JH Lyu CJ. Comprehensive clinical follow-up of late effects in childhood cancer survivors shows the need for early and well-timed intervention. Ann Oncol (2009) 20(7):1170–7. doi: 10.1093/annonc/mdn778 19270031

[19] Roughton K Kalm M Blomgren K. Sex-dependent differences in behavior and hippocampal neurogenesis after irradiation to the young mouse brain. Eur J Neurosci (2012) 36(6):2763–72. doi: 10.1111/j.1460-9568.2012.08197.x 22758785

[20] Monje M Dietrich J. Cognitive side effects of cancer therapy demonstrate a functional role for adult neurogenesis. Behav Brain Res (2012) 227(2):376–9. doi: 10.1016/j.bbr.2011.05.012 PMC3221863 21621557

[21] Monje ML Vogel H Masek M Ligon KL Fisher PG Palmer TD. Impaired human hippocampal neurogenesis after treatment for central nervous system malignancies. Ann Neurol (2007) 62(5):515–20. doi: 10.1002/ana.21214 17786983

[22] Kalm M Fukuda A Fukuda H Ohrfelt A Lannering B Bjork-Eriksson T. Transient inflammation in neurogenic regions after irradiation of the developing brain. Radiat Res (2009) 171(1):66–76. doi: 10.1667/RR1269.1 19138045

[23] Monje ML Toda H Palmer TD. Inflammatory blockade restores adult hippocampal neurogenesis. Science (2003) 302(5651):1760–5. doi: 10.1126/science.1088417 14615545

[24] Osman AM Sun Y Burns TC He L Kee N Oliva-Vilarnau N. Radiation triggers a dynamic sequence of transient microglial alterations in juvenile brain. Cell Rep (2020) 31(9):107699. doi: 10.1016/j.celrep.2020.107699 32492415

[25] Palmer JD Tsang DS Tinkle CL Olch AJ Kremer LCM Ronckers CM. Late effects of radiation therapy in pediatric patients and survivorship. Pediatr Blood Cancer (2021) 68(Suppl 2):e28349. doi: 10.1002/pbc.28349 33818893

[26] Maekawa M Namba T Suzuki E Yuasa S Kohsaka S Uchino S. NMDA receptor antagonist memantine promotes cell proliferation and production of mature granule neurons in the adult hippocampus. Neurosci Res (2009) 63(4):259–66. doi: 10.1016/j.neures.2008.12.006 19367785

[27] Volbracht C van Beek J Zhu C Blomgren K Leist M. Neuroprotective properties of memantine in different *in vitro* and *in vivo* models of excitotoxicity. Eur J Neurosci (2006) 23(10):2611–22. doi: 10.1111/j.1460-9568.2006.04787.x 16817864

[28] Marvanova M Lakso M Pirhonen J Nawa H Wong G Castren E. The neuroprotective agent memantine induces brain-derived neurotrophic factor and trkB receptor expression in rat brain. Mol Cell Neurosci (2001) 18(3):247–58. doi: 10.1006/mcne.2001.1027 11591126

[29] Rogawski MA Wenk GL. The neuropharmacological basis for the use of memantine in the treatment of alzheimer's disease. CNS Drug Rev (2003) 9(3):275–308. doi: 10.1111/j.1527-3458.2003.tb00254.x 14530799 PMC6741669

[30] Hellweg R Wirth Y Janetzky W Hartmann S. Efficacy of memantine in delaying clinical worsening in alzheimer's disease (AD): responder analyses of nine clinical trials with patients with moderate to severe AD. Int J Geriatr Psychiatry (2012) 27(6):651–6. doi: 10.1002/gps.2766 22513699

[31] Rainer M Wuschitz A Jagsch C Erb C Chirikdjian JJ Mucke HA. Memantine in moderate to severe alzheimer's disease: an observational post-marketing study. J Neural Transm (Vienna) (2011) 118(8):1255–9. doi: 10.1007/s00702-011-0623-8 21461744

[32] Takahashi K Nakagawasai O Nemoto W Kadota S Isono J Odaira T. Memantine ameliorates depressive-like behaviors by regulating hippocampal cell proliferation and neuroprotection in olfactory bulbectomized mice. Neuropharmacology (2018) 137:141–55. doi: 10.1016/j.neuropharm.2018.04.013 29729893

[33] Ishikawa R Kim R Namba T Kohsaka S Uchino S Kida S. Time-dependent enhancement of hippocampus-dependent memory after treatment with memantine: Implications for enhanced hippocampal adult neurogenesis. Hippocampus (2014) 24(7):784–93. doi: 10.1002/hipo.22270 24599753

[34] Sun D Chen J Bao X Cai Y Zhao J Huang J. Protection of radial glial-like cells in the hippocampus of APP/PS1 mice: a novel mechanism of memantine in the treatment of alzheimer's disease. Mol Neurobiol (2015) 52(1):464–77. doi: 10.1007/s12035-014-8875-6 25195698

[35] Minkeviciene R Banerjee P Tanila H. Memantine improves spatial learning in a transgenic mouse model of alzheimer's disease. J Pharmacol Exp Ther (2004) 311(2):677–82. doi: 10.1124/jpet.104.071027 15192085

[36] Liu MY Wang S Yao WF Zhang ZJ Zhong X Sha L. Memantine improves spatial learning and memory impairments by regulating NGF signaling in APP/PS1 transgenic mice. Neuroscience (2014) 273:141–51. doi: 10.1016/j.neuroscience.2014.05.011 24846616

[37] Brown PD Pugh S Laack NN Wefel JS Khuntia D Meyers C. Memantine for the prevention of cognitive dysfunction in patients receiving whole-brain radiotherapy: a randomized, double-blind, placebo-controlled trial. Neuro Oncol (2013) 15(10):1429–37. doi: 10.1093/neuonc/not114 PMC3779047 23956241

[38] Lehrer EJ Jones BM Dickstein DR Green S Germano IM Palmer JD. The cognitive effects of radiotherapy for brain metastases. Front Oncol (2022) 12:893264. doi: 10.3389/fonc.2022.893264 35847842 PMC9279690

[39] Duman JG Dinh J Zhou W Cham H Mavratsas VC Paveskovic M. Memantine prevents acute radiation-induced toxicities at hippocampal excitatory synapses. Neuro Oncol (2018) 20(5):655–65. doi: 10.1093/neuonc/nox203 PMC5892158 29112734

[40] Zhang D Zhou W Lam TT Weng C Bronk L Ma D. Radiation induces age-dependent deficits in cortical synaptic plasticity. Neuro Oncol (2018) 20(9):1207–14. doi: 10.1093/neuonc/noy052 PMC6107999 29660023

[41] Hokama Y Nishimura M Usugi R Fujiwara K Katagiri C Takagi H. Recovery from the damage of cranial radiation modulated by memantine, an NMDA receptor antagonist, combined with hyperbaric oxygen therapy. Neuro Oncol (2023) 25(1):108–22. doi: 10.1093/neuonc/noac162 PMC9825311 35762568

[42] Ben Abdallah NM Slomianka L Vyssotski AL Lipp HP. Early age-related changes in adult hippocampal neurogenesis in C57 mice. Neurobiol Aging (2010) 31(1):151–61. doi: 10.1016/j.neurobiolaging.2008.03.002 18455269

[43] Kuhn HG Dickinson-Anson H Gage FH. Neurogenesis in the dentate gyrus of the adult rat: age-related decrease of neuronal progenitor proliferation. J Neurosci (1996) 16(6):2027–33. doi: 10.1523/JNEUROSCI.16-06-02027.1996 PMC6578509 8604047

[44] Fukuda A Fukuda H Swanpalmer J Hertzman S Lannering B Marky I. Age-dependent sensitivity of the developing brain to irradiation is correlated with the number and vulnerability of progenitor cells. J Neurochem (2005) 92(3):569–84. doi: 10.1111/j.1471-4159.2004.02894.x 15659227

[45] Fowler JF. The linear-quadratic formula and progress in fractionated radiotherapy. Br J Radiol (1989) 62(740):679–94. doi: 10.1259/0007-1285-62-740-679 2670032

[46] Naylor AS Bull C Nilsson MK Zhu C Bjork-Eriksson T Eriksson PS. Voluntary running rescues adult hippocampal neurogenesis after irradiation of the young mouse brain. Proc Natl Acad Sci USA (2008) 105(38):14632–7. doi: 10.1073/pnas.0711128105 PMC2567198 18765809

[47] Ayoub R Ruddy RM Cox E Oyefiade A Derkach D Laughlin S. Assessment of cognitive and neural recovery in survivors of pediatric brain tumors in a pilot clinical trial using metformin. Nat Med (2020) 26(8):1285–94. doi: 10.1038/s41591-020-0985-2 PMC8176964 32719487

[48] Kornhuber J Quack G. Cerebrospinal fluid and serum concentrations of the n-methyl-D-aspartate (NMDA) receptor antagonist memantine in man. Neurosci Lett (1995) 195(2):137–9. doi: 10.1016/0304-3940(95)11785-U 7478269

[49] Overall RW Walker TL Fischer TJ Brandt MD Kempermann G. Different mechanisms must be considered to explain the increase in hippocampal neural precursor cell proliferation by physical activity. Front Neurosci (2016) 10:362. doi: 10.3389/fnins.2016.00362 27536215 PMC4971098

[50] Kuhn HG Cooper-Kuhn CM. Bromodeoxyuridine and the detection of neurogenesis. Curr Pharm Biotechnol (2007) 8(3):127–31. doi: 10.2174/138920107780906531 17584085

[51] Bergles DE Richardson WD. Oligodendrocyte development and plasticity. Cold Spring Harb Perspect Biol (2015) 8(2):a020453. doi: 10.1101/cshperspect.a020453 26492571 PMC4743079

[52] Tay TL Savage JC Hui CW Bisht K Tremblay ME. Microglia across the lifespan: from origin to function in brain development, plasticity and cognition. J Physiol (2017) 595(6):1929–45. doi: 10.1113/JP272134 PMC5350449 27104646

[53] Wong-Goodrich SJ Pfau ML Flores CT Fraser JA Williams CL Jones LW. Voluntary running prevents progressive memory decline and increases adult hippocampal neurogenesis and growth factor expression after whole-brain irradiation. Cancer Res (2010) 70(22):9329–38. doi: 10.1158/0008-5472.CAN-10-1854 PMC2982943 20884629

[54] Huo K Sun Y Li H Du X Wang X Karlsson N. Lithium reduced neural progenitor apoptosis in the hippocampus and ameliorated functional deficits after irradiation to the immature mouse brain. Mol Cell Neurosci (2012) 51(1-2):32–42. doi: 10.1016/j.mcn.2012.07.002 22800605

[55] Zhou K Xie C Wickstrom M Dolga AM Zhang Y Li T. Lithium protects hippocampal progenitors, cognitive performance and hypothalamus-pituitary function after irradiation to the juvenile rat brain. Oncotarget (2017) 8(21):34111–27. doi: 10.18632/oncotarget.16292 PMC5470955 28415806

[56] Zanni G Goto S Fragopoulou AF Gaudenzi G Naidoo V Di Martino E. Lithium treatment reverses irradiation-induced changes in rodent neural progenitors and rescues cognition. Mol Psychiatry (2021) 26(1):322–40. doi: 10.1038/s41380-019-0584-0 PMC7815512 31723242

[57] Devi L Ohno M. Cognitive benefits of memantine in alzheimer's 5XFAD model mice decline during advanced disease stages. Pharmacol Biochem Behav (2016) 144:60–6. doi: 10.1016/j.pbb.2016.03.002 26948858

[58] Bulin SE Mendoza ML Richardson DR Song KH Solberg TD Yun S. Dentate gyrus neurogenesis ablation *via* cranial irradiation enhances morphine self-administration and locomotor sensitization. Addict Biol (2018) 23(2):665–75. doi: 10.1111/adb.12524 PMC5775053 28626932

[59] Rivera PD Simmons SJ Reynolds RP Just AL Birnbaum SG Eisch AJ. Image-guided cranial irradiation-induced ablation of dentate gyrus neurogenesis impairs extinction of recent morphine reward memories. Hippocampus (2019) 29(8):726–35. doi: 10.1002/hipo.23071 PMC7036142 30779299

[60] Blomstrand M Kalm M Grander R Bjork-Eriksson T Blomgren K. Different reactions to irradiation in the juvenile and adult hippocampus. Int J Radiat Biol (2014) 90(9):807–15. doi: 10.3109/09553002.2014.942015 25004947

[61] Fukuda H Fukuda A Zhu C Korhonen L Swanpalmer J Hertzman S. Irradiation-induced progenitor cell death in the developing brain is resistant to erythropoietin treatment and caspase inhibition. Cell Death Differ (2004) 11(11):1166–78. doi: 10.1038/sj.cdd.4401472 15243583

[62] von Bohlen Und Halbach O von Bohlen Und Halbach V. BDNF effects on dendritic spine morphology and hippocampal function. Cell Tissue Res (2018) 373(3):729–41. doi: 10.1007/s00441-017-2782-x 29450725

[63] Pardon MC. Role of neurotrophic factors in behavioral processes: implications for the treatment of psychiatric and neurodegenerative disorders. Vitam Horm (2010) 82:185–200. doi: 10.1016/S0083-6729(10)82010-2 20472139

[64] Ketschek A Spillane M Gallo G. Mechanism of NGF-induced formation of axonal filopodia: NGF turns up the volume, but the song remains the same? Commun Integr Biol (2011) 4(1):55–8. doi: 10.4161/cib.13689 PMC3073271 21509179

[65] Zhang Y Gao F Wu D Moshayedi P Zhang X Ellamushi H. Lentiviral mediated expression of a NGF-soluble nogo receptor 1 fusion protein promotes axonal regeneration. Neurobiol Dis (2013) 58:270–80. doi: 10.1016/j.nbd.2013.06.008 23811498

[66] Danzer SC Crooks KR Lo DC McNamara JO. Increased expression of brain-derived neurotrophic factor induces formation of basal dendrites and axonal branching in dentate granule cells in hippocampal explant cultures. J Neurosci (2002) 22(22):9754–63. doi: 10.1523/JNEUROSCI.22-22-09754.2002 PMC6757848 12427830

[67] Lang UE Muhlbacher M Hesselink MB Zajaczkowski W Danysz W Danker-Hopfe H. No nerve growth factor response to treatment with memantine in adult rats. J Neural Transm (Vienna) (2004) 111(2):181–90. doi: 10.1007/s00702-003-0090-y 14767721

[68] Ficek J Zygmunt M Piechota M Hoinkis D Rodriguez Parkitna J Przewlocki R. Molecular profile of dissociative drug ketamine in relation to its rapid antidepressant action. BMC Genomics (2016) 17:362. doi: 10.1186/s12864-016-2713-3 27188165 PMC4869301

[69] Zanni G Di Martino E Omelyanenko A Andang M Delle U Elmroth K. Lithium increases proliferation of hippocampal neural stem/progenitor cells and rescues irradiation-induced cell cycle arrest in vitro. Oncotarget (2015) 6(35):37083–97. doi: 10.18632/oncotarget.5191 PMC4741917 26397227

[70] Baldessarini RJ Tondo L Vazquez GH. Pharmacological treatment of adult bipolar disorder. Mol Psychiatry (2019) 24(2):198–217. doi: 10.1038/s41380-018-0044-2 29679069

