# Supplementary figures and images for: Memantine increases the dendritic complexity of hippocampal young neurons in the juvenile brain after cranial irradiation
Source: Front Oncol. 2023 Oct 4;13:1202200. doi: 10.3389/fonc.2023.1202200 (PMC10584145; doi:10.3389/fonc.2023.1202200)

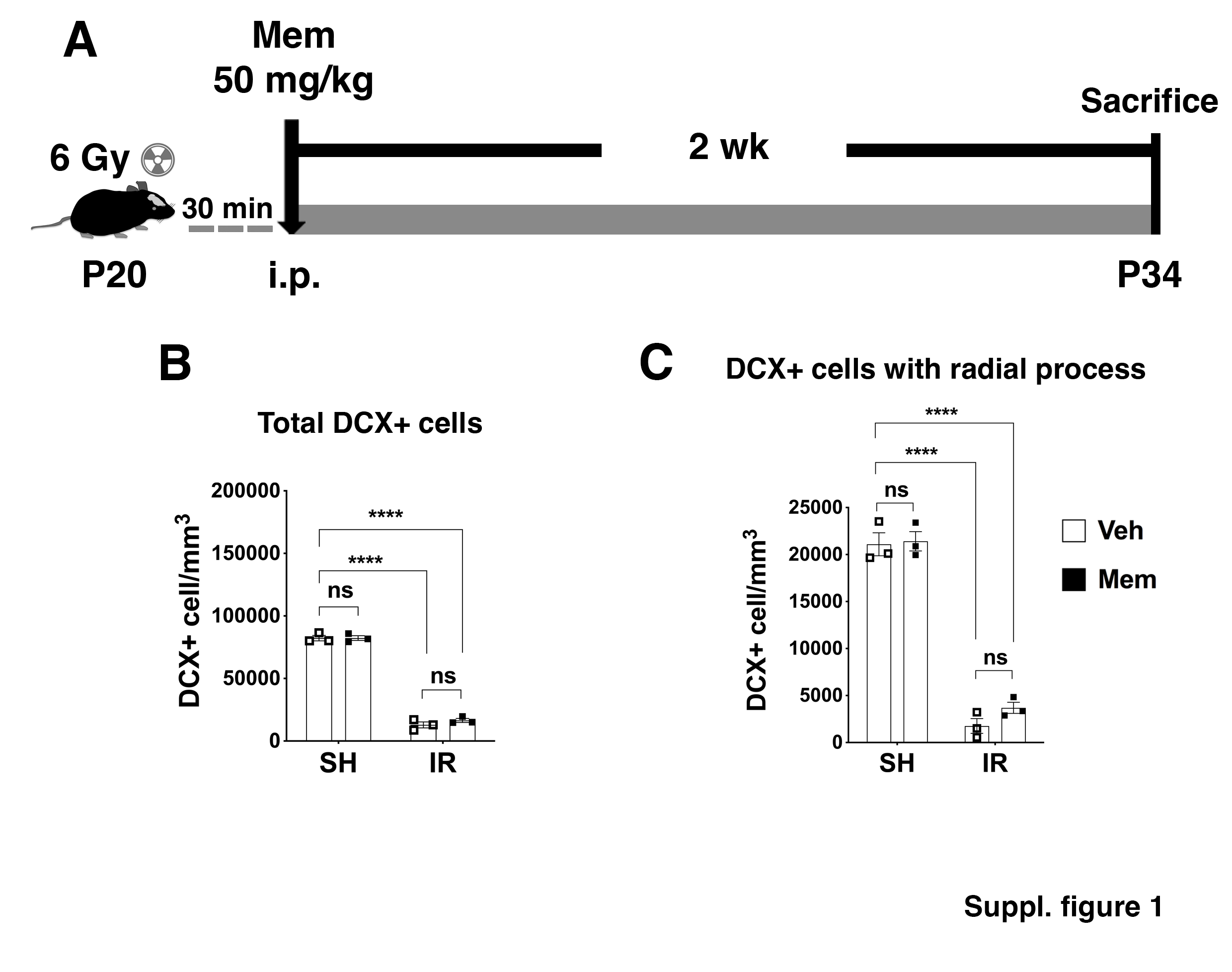

Supplement: Supplementary Figure 1 — Single i.p. injection of a high dose of Mem does not impact on the number of DCX+ cells with radial processes at 2 weeks post-treatment. (A) Scheme for the experimental design. wk = week. (B) Quantification of total DCX+ cells in the subgranular zone (SGZ) and the granule cell layer in the dentate gyrus of the treatment groups. n = 3 per group. Data represent mean ± SEM. Two-way ANOVA with Tukey’s post hoc test for multiple comparisons. ****P < 0.0001. ns = not significant. (C) Quantification of DCX+ cells with radial processes. n = 3 per group. Data represent mean ± SEM. Two-way ANOVA with Tukey’s post hoc test for multiple comparisons. ****P < 0.0001. ns = not significant. [file Image_1.tif]

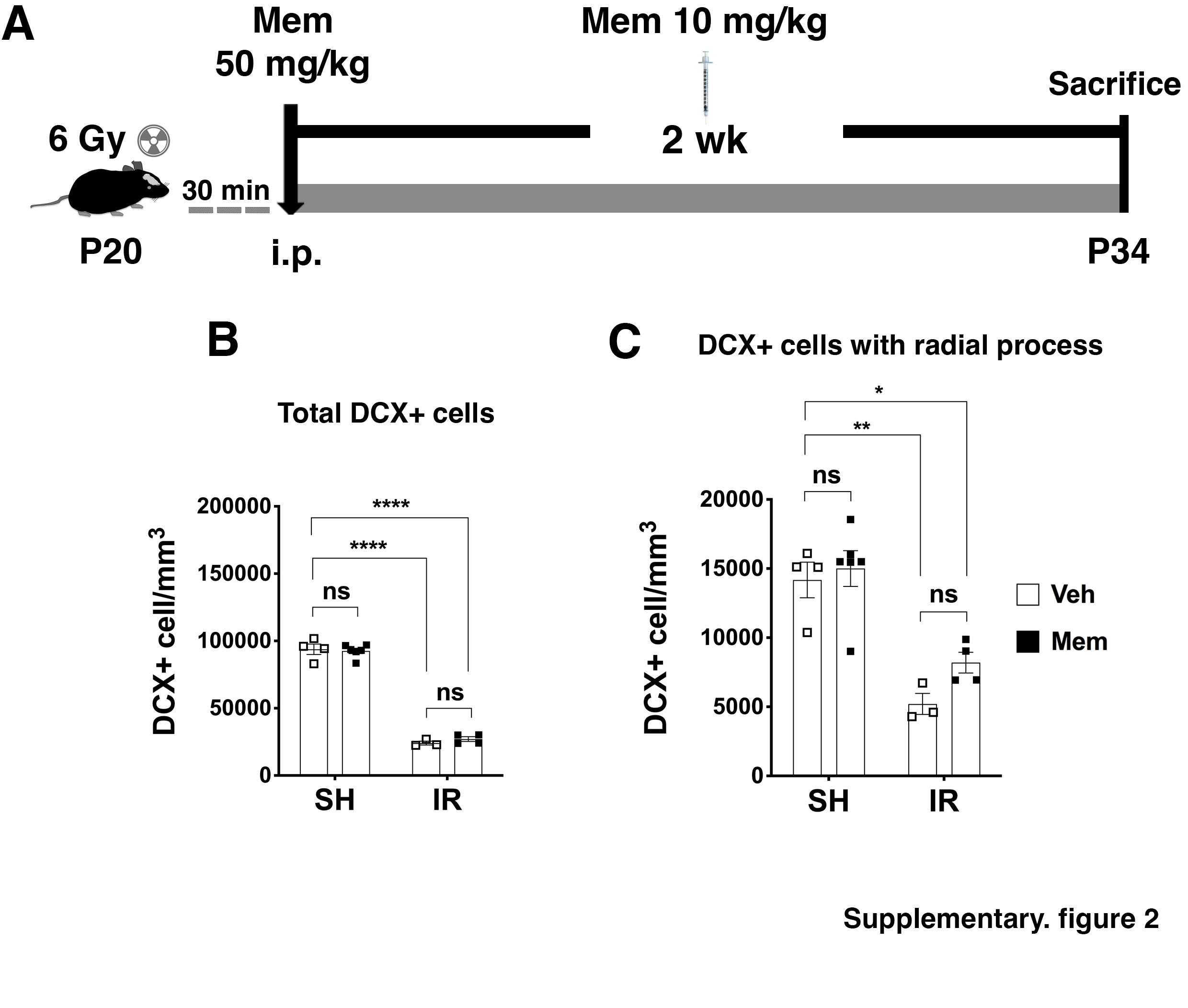

Supplement: Supplementary Figure 2 — Intermittent administration of a low does not no impact on the number of DCX+ cells with radial processes at 2 weeks post-treatment. (A) Scheme for the experimental design. wk = week. (B) Quantification of total DCX+ cells in the SGZ and the GCL in the dentate gyrus of the treatment groups. SH + Veh n = 4; SH + Mem n = 6; IR + Veh n = 3; IR + Mem n = 4. Data represent mean ± SEM. Two-way ANOVA with Tukey’s post hoc test for multiple comparisons. ****P <0.0001. ns = not significant. (C) Quantification of DCX+ cells with radial processes. n = 3-6 per treatment. Two-way ANOVA with Tukey’s post hoc test for multiple comparisons. *P < 0.03, **P < 0.005. ns = not significant. [file Image_2.tif]

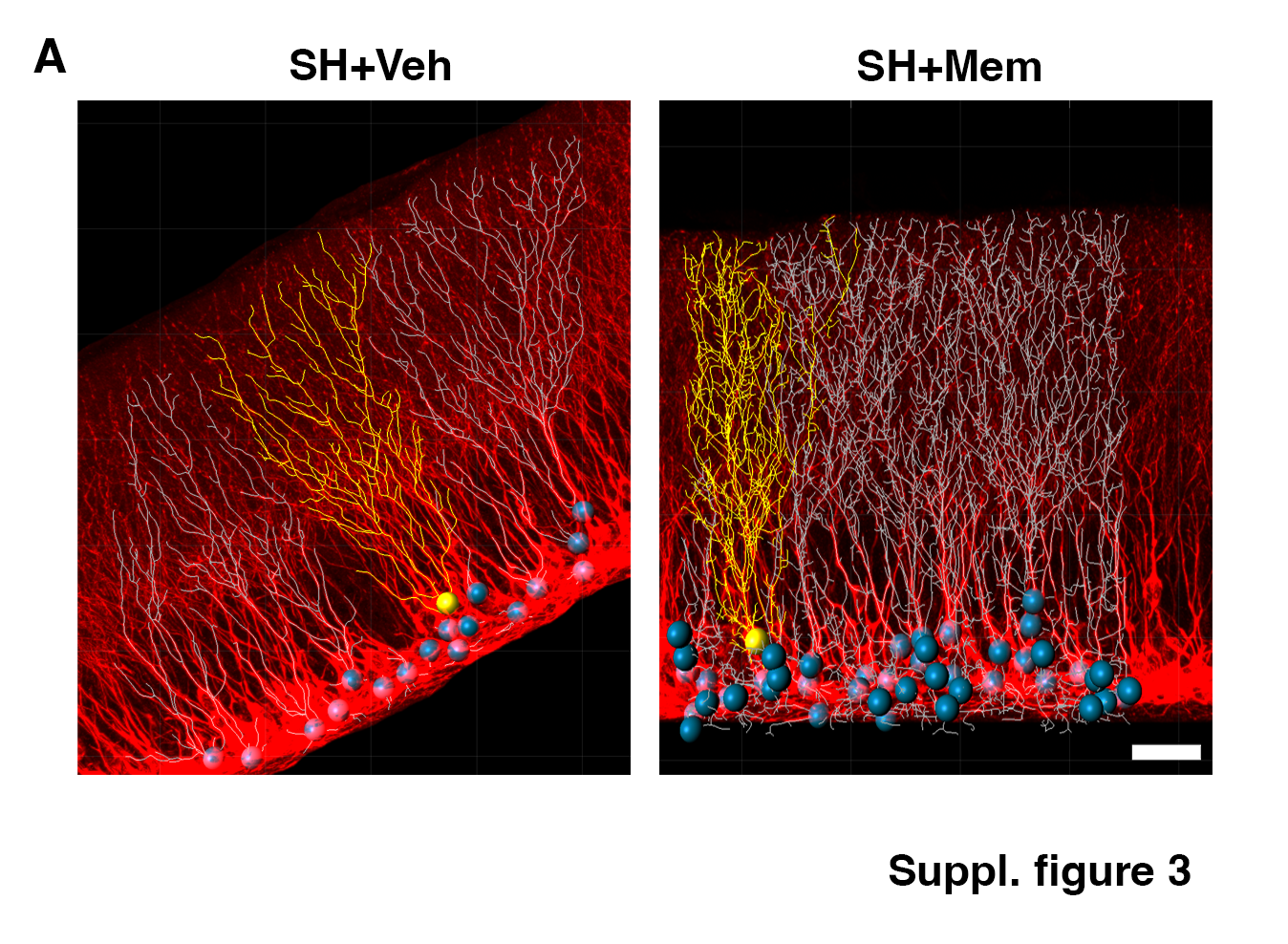

Supplement: Supplementary Figure 3 — Filament tracing of DCX+ cells in sham controls animals. (A) Representative immunofluorescent images show filament tracing of reconstructed DCX+ cells in the GCL of SH animals treated with either Veh or Mem. Accurate tracing of individual cells without an overlap with neighboring cells was challenging as demonstrated by the yellow and white tracing in each treatment group. Scalebar = 30 μm. [file Image_3.tif]
